# Density of outdoor advertising of consumable products in NYC by neighborhood poverty level

**DOI:** 10.1186/s12889-019-7821-y

**Published:** 2019-11-08

**Authors:** Tamar Adjoian, Rachel Dannefer, Shannon M. Farley

**Affiliations:** 1 New York City Department of Health and Mental Hygiene, Bureau of Chronic Disease Prevention, 42-09 28th Street, Long Island City, NY 11101 USA; 2 New York City Department of Health and Mental Hygiene, Bureau of Harlem Neighborhood Health at the East Harlem Neighborhood Health Action Center, 161-169 East 110th Street, New York, NY 10029 USA

**Keywords:** Advertising, Urban, Chronic disease, Tobacco control, Retail environment

## Abstract

**Background:**

To determine if outdoor advertising density for non-alcoholic drinks, food, tobacco products, and alcohol, is associated with neighborhood poverty or other Census-level characteristics in New York City (NYC).

**Methods:**

From June – July of 2015, photographs were taken of all street-level, stationary outdoor advertising (posters, stickers, decals, etc.) for consumable products in a sample of 953 NYC retail-dense street segments. Density of product images was analyzed by neighborhood poverty level and other characteristics using multivariate negative-binomial regression.

**Results:**

A total of 16,305 discrete advertisements displaying 50,673 product images were photographed. Total product image prevalence relative to retail density was not significantly higher in high- vs. low-poverty neighborhoods, as hypothesized (OR: 1.31; 95% CI: 0.98, 1.77). However, total product image prevalence was higher in neighborhoods with a higher percentage of Black residents (OR: 1.08; 95% CI: 1.04, 1.12), and for sugary drinks in areas with a higher percentage of adults with <HS diploma (OR: 1.32; 95% CI: 1.11, 1.58).

**Conclusions:**

Product images were abundant throughout NYC’s retail-dense areas, with marginally greater prevalence by some Census-level demographics, irrespective of the content displayed.

## Background

Environments play a key role in the proliferation or prevention of chronic disease, whether by promoting less healthy or healthier choices [1]. Contextual factors, like advertising, can influence purchase behavior. Consequently, when advertising is for unhealthy products (e.g., sugary drinks, tobacco products, and alcohol), it can contribute to consumption of these products [2–8], which are associated with a number of chronic diseases and negative health outcomes [9]. Understanding the physical environment and its cues that encourage individuals to make counter-intuitive choices due to unconscious instincts [2], is an emerging area in the field of public health.

From a health equity perspective, the distribution of such cues is important, since the influence of advertising may not always be felt equally—several studies have demonstrated a disproportionate amount of advertising of unhealthy products to specific populations, including communities of color, people with low incomes and those with lower educational attainment, and children [10–16]. A more detailed review of extant literature is presented in Additional file 1.

In New York City (NYC), residents of high-poverty neighborhoods (> 20% of residents living below the Federal Poverty Level (FPL)) are more likely to consume one or more sugary drinks per day than those in low-poverty neighborhoods (< 10% of residents living below the FPL) (25% vs. 16%, *p* < .001) and to be a current smoker (14% vs. 11%, *p* = .026). Additionally, chronic conditions related to these behaviors are more common in high-poverty neighborhoods, with increased prevalence of obesity, diabetes, and hypertension compared to low-poverty areas (obesity: 28% vs. 20%, *p* < .001; diabetes: 13% vs. 8%, *p* < .001; hypertension: 31% vs. 24%, *p* < .001) [17].

In NYC, there is also a clear relationship between neighborhood poverty and race/ethnicity; a disproportionate percentage of Black and Latino residents live in high-poverty neighborhoods, and a higher percentage of White residents live in low-poverty areas [18].

This study was designed to estimate the density of outdoor advertising in NYC overall and in low-, medium- and high-poverty neighborhoods for four target product categories: (1) non-alcoholic beverages (such as sugary drinks, water, coffee, etc.); (2) food products (including fresh produce, sweets, and other food); (3) tobacco products (including tobacco-containing products as well as electronic nicotine delivery systems (ENDS) such as e-cigarettes); and (4) alcoholic beverages; these four product categories of interest will hereafter be referred to as “consumable products.” Our primary outcomes of interest were the number of advertisements and product images featured in advertisements on retail-dense street segments for these target products, both citywide and in high-, medium-, and low-poverty neighborhoods. To our knowledge, this study is the first to collect data on outdoor, street-level advertising of such a variety of consumable products in the densely-populated urban environment of New York City.

## Methods

The comprehensive methodology for this study has been described in Additional file 1. In short, we first defined “retail-dense” areas of the city as street segments (i.e., city blocks, including both sides of the street) with retail establishments occupying at least 50% of total establishments based on existing administrative data from 2014 to 2015 (see Additional file 1: Figure S1). A random sample of retail-dense street segments was selected from eligible segments, stratified by neighborhood-level poverty (low, medium, and high) and NYC borough (the Bronx, Brooklyn, Manhattan, Queens, and Staten Island). Using NYC Department of Health and Mental Hygiene (Health Department) guidance to define neighborhood poverty [19], street segments were considered low-poverty if fewer than 10% of residents lived below the FPL, medium-poverty if 10 to < 20% of residents lived below the FPL, and high-poverty if 20% or more of residents lived below the FPL. Poverty rates were calculated using census tract-level income information from the 2009–2013 American Community Survey 5-year estimates [20].

The target sample size was 1050 street segments in the 15 strata (5 boroughs × 3 poverty levels). Proportional sampling was used to randomly sample street segments within each stratum, ensuring that each had at least 50 street segments to allow for comparisons between strata; remaining street segments were distributed proportionally into strata. In addition, we oversampled each stratum by 5% in case segments were deemed ineligible upon visiting (e.g., because they were inaccessible or lacking retail), giving us a total of 1106 street segments in the original sample, with the goal of completing data collection on at least 1050 total segments.

Our definition for advertisements was informed by a variety of sources. Advertisements included in this study were street-level, stationary signs (posters, stickers, decals, digital signs, etc.) [12, 14, 21] that displayed a product with the intended purpose of promoting that product or type of product [22, 23]. One ad was considered the discrete, physical unit of the poster, sticker, decal etc., even if multiple products were featured. Additional criteria for defining advertisements are outlined in Additional file 1: Table S2.

Data collection occurred between June 22nd and July 22nd, 2015. Photographs were taken of street-level advertisements only (from the ground up to awnings) to reflect how they would be seen by pedestrians walking down the sidewalk on a given street segment. Ads were photographed with sufficient detail to identify the address and location type of the ad placement (e.g., restaurant, corner store, phone booth, etc.). Digital ads were captured if the content featured consumable products; if they cycled through multiple advertisements, only one out of the rotation was photographed, whichever was displayed while the photo was being taken.

Following data collection, photographs were coded for location type and content. For each ad, coders noted the presence of the following content: (1) non-alcoholic beverages, including sugary drinks, low-calorie drinks, water or seltzer, unsweetened coffee, other drinks, and unknown drinks; (2) food, including fresh produce, sweets, and other, a notation was also made if ads were for fast food; (3) tobacco, including cigarettes and other tobacco products, paraphernalia (such as rolling papers, hookah pipes, etc.), and ENDS, including e-cigarettes, vape pens, and items included in ENDS kits; (4) alcoholic beverages, including beer, wine, wine products, alcopops, malt beverages, malt liquor, hard liquor, etc.; (5) branded products, with familiar and widely-recognized logos; (6) child-directed marketing, featuring cartoon characters, popular movie, TV, or sports figures, or other deliberate appeal to children [24, 25]; (7) violent or degrading imagery, featuring threatening or sexual treatment of people [26–28]; and (8) NYC Health Department advertising, featuring public service messages related to consumable products. Detailed definitions and examples of each of these coding designations are available in Additional file 1: Table S3.

When ads contained multiple products, the number of distinct images and types of products advertised was counted. Identical products that appeared multiple times in the same ad were not each counted as distinct images. For example, one ad with all the following images would be said to contain three distinct product images: a six pack of beer, two identical hamburgers, and two identical soda bottles. If different varieties of one type of product appeared in an ad, including different sizes or flavors of packaged products, each of these counted as a distinct product image. For example, an ad with a root beer and a cola bottle would count as 2 product images for sugary drinks. Since the size of individual advertisements was not captured, the number of product images within ads was used as a proxy for the extent of advertising exposure and in turn, product images were used as the primary unit of analysis.

### Statistical analysis

Descriptive statistics were used to summarize street segment, advertisement, and product image counts. Negative binomial regression was used to explore differences in product image density by neighborhood poverty level. Additional population characteristics were also available from the Census data, including educational attainment, age distribution, and racial/ethnic composition. Because previous studies have shown associations between such characteristics and advertising for consumable products [10–16, 26, 29], these variables were retained to include as covariates in analyses. Age groups were collapsed into % under 18 and % 18 and up, education was collapsed into % with less than a high school (HS) diploma and % with a HS diploma or higher, and race/ethnicity was presented as % of each Census tract that was Black, Hispanic, Asian, White, or Other, as categorized by the U.S. Census Bureau [20]. Bivariate models were run for each covariate to determine significance; however, % of White residents was excluded due to high correlation with other race/ethnicity variables. Multivariate models using proc. glimmix included significant covariates, as well as NYC borough, which was used to define the sampling strata. As described previously, retail-dense street segments were defined as having at least 50% of total establishments occupied by retailers, with a minimum of 4 store entrances per segment. The planned sample size of 1050 street segments provided 80% power to detect a difference of 8 to 10 percentage points between categories of advertising content in high- versus low-poverty neighborhoods. To account for the variation in the number of retailers per segment, the natural logarithm of the number of store entrances per segment was used as an offset variable (hereafter referred to as “retail density”). Exponentiated parameter estimates were computed to determine the odds of increased/decreased volume of product images relative to retail density by poverty level and for each increase of 10 percentage points in other covariates. All analyses were conducted using SAS Enterprise Guide version 7.1 (SAS Institute Inc., Cary, NC) with α = .05, and all comparisons referenced in text are *p* < .05 unless otherwise stated. This project was submitted to the Health Department’s Institutional Review Board and was determined to not be human subjects research.

## Results

Of the 1106 sampled segments, 953 (86%) were eligible and 153 (14%) were ineligible (*n* = 98 with no entrances to retail stores, *n* = 31 at least 50% covered by scaffolding, *n* = 14 with stores/retail entrances substantially setback from the sidewalk, *n* = 10 otherwise inaccessible) (see Additional file 1: Figure S2). Eligible street segments were fairly evenly distributed across neighborhood poverty levels, with 32% in low-poverty, 33% in medium-poverty, and 35% in high-poverty neighborhoods, and retail density of street segments did not vary much by poverty level, with a mean of 11–12 retail doorways per segment. Socio-demographic characteristics of the eligible segments overall and by neighborhood poverty level, are presented in Table 1.

**Table 1:** Census Tract Socio-Demographic Characteristics, American Community Survey 5-year estimates, 2009–2013, NYC Community Marketing Study, 2015

	Overall			Low Poverty			Medium Poverty			High Poverty		
	Median	Lower Quartile	Upper Quartile	Median	Lower Quartile	Upper Quartile	Median	Lower Quartile	Upper Quartile	Median	Lower Quartile	Upper Quartile
Population	3904	2879	5604	3688	2511	5404	3591	2818	5138	4556	3298	6031
Race/Ethnicity^a^												
White (%)	34.2	6.1	66.5	67.5	46.8	78.5	39.2	9.7	63.2	9.0	2.9	25.8
Black (%)	4.8	1.4	27.7	2.7	0.8	7.2	4.0	1.2	21.5	18.8	3.1	41.4
Latino (%)	17.4	9.1	40.4	10.0	6.4	17.2	16.7	9.4	31.9	39.6	17.4	64.6
Asian (%)	8.2	2.5	18.1	8.7	4.0	16.4	11.7	5.3	26.7	3.8	1.4	12.5
Age												
Under 18 (%)	20.4	15.3	25.2	16.9	11.6	21.1	19.4	15.0	22.6	25.2	19.8	30.6
Education												
< High School (%)	17.5	9.3	28.2	8.0	3.2	13.6	16.7	11.0	22.6	30.3	22.5	39.3

^a^ Latino includes persons of Hispanic or Latino origin regardless of reported race. Black, White and Asian race categories exclude those who identified as Hispanic or Latino. Participants identifying as American Indian, Alaska Native, Pacific Islander, or Other were excluded from this table

### Advertisements

There were a total of 16,305 discrete advertisements for consumable products. The average street segment was 314 ft long (approx. 96 m), and the average number of ads per street segment was 17 (range: 0–150, IQR: 3–24). One-hundred forty-two segments had no advertising for consumable products (13%). Over one fourth of all ads featured other food or sugary drinks (29 and 27%, respectively), 20% featured alcohol, 10% featured tobacco products and/or ENDS, 9% featured sweets, and 9% featured fresh produce (Table 2).

**Table 2:** Advertisements Featuring Various Products and Total Product Counts, Unadjusted Percentages, NYC Community Marketing Study, 2015

Product Count Category	Total Ads^a^		Total Product Count	
	N	% of total	N	% of total
Overall	**16,305**	**100.0%**	**50,673**	**100.0%**
Non-Alcoholic Beverages	**6294**	**38.6%**	**12,125**	**23.9%**
Sugary Drinks^b^	4497	27.6%	8197	16.2%
Low Calorie Drinks	430	2.6%	644	1.3%
Water/Seltzer	321	2.0%	391	0.8%
Unsweetened Coffee	735	4.5%	944	1.9%
Other Drinks	863	5.3%	1472	2.9%
Unknown Drinks	252	1.5%	477	0.9%
Food	**6814**	**41.8%**	**27,218**	**53.7%**
Fast Food	590	3.6%	1397	2.8%
Fresh Produce	1480	9.1%	6970	13.8%
Sweets	1432	8.8%	4656	9.2%
Other Food	4756	29.2%	15,592	30.8%
Tobacco/ENDS^c^ Products	**1680**	**10.3%**	**3726**	**7.4%**
Tobacco	1077	6.6%	1755	3.5%
ENDS	618	3.8%	1971	3.9%
Alcohol	**3334**	**20.4%**	**7604**	**15.0%**

^a^ Categories are not mutually exclusive; columns will not sum to 100%

^b^ Sugary drinks include coffee drinks and dairy-based drinks with added sugar

^c^ ENDS are electronic nicotine delivery systems

In unadjusted analyses, high-poverty neighborhoods had 20 ads per street segment on average (range 0–120), compared with 18 in medium-poverty areas (range 0–150) and 13 in low-poverty areas (range 0–93) (low- vs. medium-poverty: *p* = .001; low- vs high-poverty: *p* < .001). This was also reflected in the disproportionate distribution of ads high-poverty neighborhoods as compared to the relatively even distribution of segments which were samples across all three income groups. Ads in these high-poverty areas accounted for 42% of all ads counted in this study (*n* = 6790 ads), compared with 35% in medium-poverty neighborhoods, and 24% in low-poverty neighborhoods.

### Product images

A total of 50,673 product images were identified in this study, with an average of 53 images per segment (range 0–536). Similarly to our findings for advertisements, unadjusted analyses showed that high-poverty neighborhoods experienced the highest saturation of product images. Distribution of product images differed by neighborhood poverty, with 22,120 in high-poverty neighborhoods (44%), 16,675 in medium-poverty neighborhoods (33%), and 11,878 in low-poverty neighborhoods (23%). Food made up more than half of product images overall (54%), followed by non-alcoholic beverages (24%), alcohol (15%) and tobacco/ENDS products (7%). Within the food category, the most prevalent subcategories of product images were “other” food (31%), followed by sugary drinks (16%) and fresh produce (14%). Within the non-alcoholic beverage category, sugary drinks comprised more than two-thirds of product images (68%; *n* = 8197), while low calorie drinks and water/seltzer combined accounted for 9% (*n* = 1035). Unsweetened coffee, “other” drinks, and unknown drinks made up the remaining 24% (*n* = 2893) (Table 2).

Adjusted negative-binomial regression results showed that, relative to retail density, prevalence of total product images was higher in medium-poverty neighborhoods compared to low poverty (OR: 1.26, 95% CI: 1.00, 1.59). Differences by neighborhood income were also seen for tobacco/ENDS (OR high- vs. low-poverty: 2.01, 95% CI: 1.23, 3.27; OR low- vs. medium-poverty: 1.79, 95% CI: 1.16, 2.76) and alcoholic beverage product images (OR low- vs. high-poverty: 1.47, 95% CI: 1.02, 2.14; OR low- vs. medium-poverty: 1.92, 95% CI: 1.38, 2.67).

Prevalence of product images also differed by population characteristics. For every 10 percentage point increase in proportion of Black residents, there was an increase in the number of total product images (OR: 1.08, 95% CI: 1.04, 1.12), food images (OR: 1.06, 95% CI: 1.01, 1.10), and non-alcoholic beverage images (OR: 1.18, 95% CI: 1.12, 1.23) relative to retail density. Conversely, for every 10 percentage point increase in the proportion of Asian residents, there was a decrease in the number of tobacco/ENDS product images (OR: 0.88, 95% CI: 0/78, 0.99) and alcoholic beverage product images (OR: 0.91, 95% CI: 0.84, 0.99) relative to retail density (Table 3). Density of product images for all non-alcoholic beverages was also higher in areas with a higher percentage of adults with less than a HS diploma (OR: 1.31, 95% CI: 1.12, 1.53).

**Table 3:** Prediction of Advertised Product Image Density relative to Retail Density, NYC Community Marketing Study, 2015

Census-level Demographics^a^	All Products		Food		Non-Alcoholic Beverages (all)		Tobacco/ENDS		Alcohol	
	Unadjusted	Adjusted	Unadjusted	Adjusted	Unadjusted	Adjusted	Unadjusted	Adjusted	Unadjusted	Adjusted
Neighborhood Poverty										
Low	ref	ref	ref	ref	ref	ref	ref	ref	ref	ref
Medium	**1.36 (1.09, 1.70)**	**1.26 (1.00, 1.59)**	1.20 (0.93, 1.54)	1.13 (0.86, 1.48)	**1.37 (1.06, 1.77)**	1.07 (0.83, 1.40)	**1.63 (1.06, 2.50)**	**1.79 (1.16, 2.76)**	**1.90 (1.38, 2.61)**	**1.92 (1.38, 2.67)**
High	**1.77 (1.43, 2.20)**	1.31 (0.98, 1.77)	**1.72 (1.34, 2.20)**	1.34 (0.95, 1.88)	**1.83 (1.42, 2.35)**	0.88 (0.62, 1.24)	**1.98 (1.30, 3.02)**	**2.01 (1.23, 3.27)**	**1.76 (1.29, 2.41)**	**1.47 (1.02, 2.14)**
Population Traits										
% Under 18	1.10 (0.99, 1.22)		1.05 (0.93, 1.19)		**1.31 (1.15, 1.50)**	0.95 (0.80, 1.12)	**1.26 (1.01, 1.57)**	0.91 (0.68, 1.23)	0.92 (0.79, 1.08)	
% < HS Diploma	**1.18 (1.10, 1.27)**	1.08 (0.97, 1.21)	**1.16 (1.07, 1.26)**	1.10 (0.97, 1.25)	**1.31 (1.20, 1.43)**	**1.31 (1.12, 1.53)**	1.14 (0.99, 1.32)		1.08 (0.98, 1.20)	
Race/Ethnicity^b^										
% Black	**1.09 (1.05, 1.13)**	**1.08 (1.04, 1.12)**	**1.07 (1.03, 1.12)**	**1.06 (1.01, 1.10)**	**1.18 (1.13, 1.23)**	**1.18 (1.12, 1.23)**	**1.11 (1.04, 1.20)**	1.07 (0.99, 1.16)	1.00 (0.95, 1.05)	
% Latino	**1.06 (1.02, 1.11)**	1.02 (0.96, 1.08)	**1.05 (1.00, 1.10)**	0.99 (0.93, 1.05)	**1.07 (1.02, 1.12)**	1.02 (0.95, 1.11)	1.06 (0.97, 1.15)		**1.10 (1.03, 1.17)**	1.07 (0.99, 1.14)
% Asian	0.95 (0.91, 1.00)		0.99 (0.93, 1.05)		**0.92 (0.87, 0.98)**	1.00 (0.92, 1.09)	**0.87 (0.78, 0.96)**	**0.88 (0.78, 0.99)**	**0.92 (0.84, 0.99)**	**0.91 (0.84, 0.99)**

^a^Estimates for all percentage variables are per 10% unit increases

^b^Latino includes persons of Hispanic or Latino origin regardless of reported race. Black, White and Asian race categories exclude those who identified as Hispanic or Latino. Participants identifying as American Indian, Alaska Native, Pacific Islander, or Other were excluded from this table

Statistically significant results are in boldface type

### Unhealthy and healthy subcategories

Non-alcoholic beverages and food also had subcategories; for non-alcoholic beverages, we considered sugary drinks to universally be “unhealthy” and water/seltzer to be “healthy,” and for food, we considered sweets to be unhealthy, while fresh fruits and vegetables were healthy. Adjusted analyses of these product subcategories showed similar patterns between healthy and unhealthy products. Product images for both sugary drinks *and* water/seltzer were more densely advertised in neighborhoods with a higher proportion of Black residents (OR sugary drinks: 1.21, 95% CI: 1.15, 1.28; OR water/seltzer: 1.16, 95% CI: 1.07, 1.26). Sugary drink product images were also more prevalent in areas with a higher proportion of residents with less than a HS education (OR: 1.32, 95% CI: 1.11, 1.58) (Table 4). Images of sweets were more prevalent in medium- versus low-poverty neighborhoods (OR: 1.51, 95% CI: 1.03, 2.22) and marginally so in areas with a higher proportion of Asian residents (OR: 1.09, 95% CI: 1.00, 1.19), while fruits and vegetable images were marginally more common in areas with a higher proportion of Black residents (OR: 1.06, 95% CI: 1.00, 1.12).

**Table 4:** Prediction of Advertised Product Image Density per Retail Doorway, NYC Community Marketing Study, 2015

	Unhealthy Products				Healthy Products			
	Sugary Drinks^b^		Sweets		Water/Seltzer		Fresh Fruits/Vegetables	
	Unadjusted	Adjusted	Unadjusted	Adjusted	Unadjusted	Adjusted	Unadjusted	Adjusted
Neighborhood Poverty								
Low	ref	ref	ref	ref	ref	ref	ref	ref
Medium	**1.38 (1.03, 1.84)**	1.06 (0.79, 1.42)	**1.63 (1.14, 2.34)**	**1.51 (1.03, 2.22)**	**1.73 (1.04, 2.90)**	1.56 (0.90, 2.71)	**1.05 (0.75, 1.49)**	0.95 (0.65, 1.38)
High	**1.89 (1.42, 2.51)**	0.82 (0.56, 1.21)	**2.06 (1.45, 2.93)**	1.60 (0.97, 2.64)	**2.33 (1.41, 3.84)**	1.42 (0.72, 2.78)	**1.64 (1.17, 2.31)**	1.11 (0.70, 1.75)
Population Traits								
% Under 18	**1.50 (1.29, 1.75)**	1.00 (0.83, 1.20)	1.00 (0.84, 1.19)		0.93 (0.73, 1.17)		1.14 (0.96, 1.35)	
% < HS Diploma	**1.39 (1.25, 1.54)**	**1.32 (1.11, 1.58)**	**1.23 (1.11, 1.37)**	1.12 (0.95, 1.32)	**1.25 (1.06, 1.48)**	1.17 (0.94, 1.46)	**1.17 (1.06, 1.30)**	1.06 (0.89, 1.27)
Race/Ethnicity^a^								
% Black	**1.21 (1.16, 1.27)**	**1.21 (1.15, 1.28)**	1.00 (0.94, 1.06)		**1.19 (1.10, 1.28)**	**1.16 (1.07, 1.26)**	**1.06 (1.00, 1.12)**	**1.06 (1.00, 1.12)**
% Latino	**1.08 (1.03, 1.15)**	1.05 (0.96, 1.15)	0.99 (0.93, 1.06)		1.02 (0.93, 1.13)		**1.09 (1.03, 1.16)**	1.05 (0.95, 1.16)
% Asian	**0.90 (0.85, 0.96)**	1.02 (0.92, 1.12)	**1.13 (1.05, 1.22)**	**1.09 (1.00, 1.19)**	**0.87 (0.77, 0.99)**	0.95 (0.82, 1.09)	0.92 (0.84, 1.00)	

^a^ Latino includes persons of Hispanic or Latino origin regardless of reported race. Black, White and Asian race categories exclude those who identified as Hispanic or Latino. Participants identifying as American Indian, Alaska Native, Pacific Islander, or Other were excluded from this table

^b^ Sugary drinks include coffee drinks and dairy-based drinks with added sugar

Statistically significant results are in boldface type

### Other ad characteristics

Aside from consumable product content, several other characteristics were documented at the advertisement level. Overall, most advertisements featured branded products (70%, *n* = 11,399), about 4% of ads had degrading or violent imagery (*n* = 583), less than 1 % of ads had public service content developed by the Health Department (*n* = 39), and less than 1 % were child focused (*n* = 48). Almost half of ads were located on bodegas or corner stores (49%), followed by restaurants (25%), supermarkets (7%), and liquor stores (6%). The vast majority of ads were in the form of posters, stickers, or signs (88%), followed by awnings (5%) and neon lights (5%). Banners and “other” made up the remainder (3%) (see Additional file 1: Table S4).

## Discussion

We found that, regardless of content, the retail environment is saturated with excessive cues to consume, whether it be food, non-alcoholic beverages, tobacco/ENDS products, or alcohol. We documented a total of 50,673 product images; of these, nearly half (48%) were for decidedly unhealthy products (sugary drinks, sweets, tobacco/ENDS and alcohol), while only 15% were for healthier products (fresh produce and water/seltzer). This supports the findings of Lowery et al. (2014) [26], which demonstrated that ads with harmful content represented about one quarter of outdoor advertising space in several communities in Los Angeles. Given that New York City is the most densely-populated major U.S. city, with more than 27,000 residents per square mile [30], it is understandable that there is high retail density as well. However, our findings show how difficult it is to avoid constant exposure to consumption cues while interacting with the retail environment. With an average of 53 images per two-sided street segment, this means that a New Yorker walking down one side of a typical retail-dense street segment would encounter roughly 26 to 27 advertised consumable product images.

Our findings build upon other research, which has demonstrated that advertising of unhealthy products is often disproportionately directed toward communities of color, people with low incomes and those with lower educational attainment, and children [10–16]. Unlike the findings of Isgor et al. (2016) [15], which showed significantly higher odds of displaying ads for unhealthy products (e.g, soda) in low- versus high-income community stores, our results did not universally support our hypothesis that advertisement density would be higher in high- versus low-poverty neighborhoods. However, we did find a higher concentration of product images to correspond to poverty level in unadjusted and some adjusted analyses. One key finding of our study was that percentage of Black residents was the most common predictor of higher product image prevalence; although ORs were small, many were statistically significant. In adjusted analyses, areas with a higher percentage of Black residents tended to have higher prevalence of overall product images, as well as images for food, non-alcoholic beverages, sugary drinks, water/seltzer, and fruits and vegetables. Although the pattern of more marketing in areas with a higher percentage of Black residents was true for healthy as well as unhealthy products, healthier products comprised a relatively small proportion of total product images. This finding indicates that Black residents in NYC likely have higher exposure to product images than other groups, including images of unhealthy products, like sugary drinks. Another analysis of this data set, which focused on advertisements of sugary drinks by NYC borough, found similar patterns [31]. These results are complementary to those of Yancey et al. (2009) [12], who looked at advertising density of high-calorie/low-nutrient products such as fast food, sugary drinks, and alcohol in several large US cities. The researchers found that predominantly African American neighborhoods had the highest ad density, followed by Latino neighborhoods; white neighborhoods had the lowest ad density.

These disparities should not be ignored, as they may be reflective of larger societal injustice. Historically, disinvestment and structural racism have resulted in neighborhoods in NYC with high poverty and limited health-promoting assets. In turn, poor health outcomes have tended to cluster in communities of color and where many residents live in poverty [32]. In the U.S., advertising is a First Amendment right, including for businesses and corporations, so there are limitations on what kind of restrictions on advertising are possible. However, public health advocates have proposed policy solutions, specifically to protect children from exposure to harmful advertising, including limiting the amount of store window space that can be covered by signs [33]. However, these types of measures may be challenging to implement and could be met with significant opposition from retailers and industry. More practicable options to counteract the immensity of unhealthy advertising could include requiring warnings on advertisements for harmful products and increased counter-marketing to promote more healthful messaging.

Our study had some limitations. First, we excluded any advertisements outside of the “street level” area (such as indoor, subway, billboards, mobile/non-stationary), so not every possible advertisement was documented. Second, we were unable to classify the majority of food images beyond fresh produce and sweets. The majority of food imagery was too complex to code more granularly. For example, many items were mixed dishes which often featured multiple components and/or had indecipherable ingredients. Therefore, the most commonly advertised consumable product category ended up being a composite of healthy and unhealthy items. Likewise, fast food imagery was not coded by specific content, even though fast food restaurants can vary widely in their food offerings. Some offer burgers, fries, pizza, fried chicken, ice cream, etc., while others offer mostly customizable salads and other healthier items. However, it could be argued that the sheer magnitude of food imagery is contributing to the obesogenic environment, continually reminding individuals to consume. Finally, we did not measure the size of advertisements, so ads were all counted as equivalents, regardless of size. However, individual product images were counted separately, which may have provided a proxy for size, since larger ads could likely contain more product images than smaller ads.

This study had several strengths to note as well. Our large sample size enabled us to conduct citywide analyses, as well as comparisons between neighborhood poverty levels and by available demographic characteristics. The methodology was very comprehensive, drawing relevant features from a variety of similar studies and incorporating several data sources from which the best possible sampling frame was generated. Finally, to our knowledge, this is the first study to look at outdoor advertising on a citywide level in NYC, including a wide range of products (non-alcoholic beverages, food, tobacco, and alcohol).

## Conclusions

We found significantly higher density of consumable product images in medium- versus low-poverty neighborhoods, and in areas with a higher proportion of Black residents, for both unhealthy and healthy products. While we were not able to link this to specific health outcomes, it is clear that there is a high density of marketing overall in the city with some areas experiencing more. This provides quantified evidence that can be used going forward to begin to modify the advertising environment at the neighborhood level in order to change the influences people receive at home, work and play.

## Supplementary information

**Additional file 1.** In-depth methodology: Density of outdoor advertising of consumable products in NYC by neighborhood poverty level. This file gives more extensive methodology of the sampling and data collection processes related to the study.

## Data Availability

The datasets used and analysed during the current study are available from the corresponding author on reasonable request.

## Abbreviations

CI: Confidence interval
ENDS: Electronic nicotine delivery systems
FPL: Federal Poverty Level
Health Department: NYC Department of Health and Mental Hygiene
HS: High school
IQR: Interquartile range
NYC: New York City
OR: Odds ratio
