# Supplementary material for: Density of outdoor advertising of consumable products in NYC by neighborhood poverty level
Source: BMC Public Health. 2019 Nov 8;19:1479. doi: 10.1186/s12889-019-7821-y (PMC6864992; doi:10.1186/s12889-019-7821-y)
Supplement: Supplementary file 1 — Additional file 1. In-depth methodology: Density of outdoor advertising of consumable products in NYC by neighborhood poverty level. This file gives more extensive methodology of the sampling and data collection processes related to the study. [file 12889_2019_7821_MOESM1_ESM.docx]

**Additional File 1:** In-depth methodology: Density of outdoor advertising of consumable products in NYC by neighborhood poverty level

**Authored by:** Tamar Adjoian, MPH; Rachel Dannefer, MPH, MIA; Calpurnyia Roberts, PhD; Susan Resnick, MA, LMSW; Gretchen Culp, PhD, MS; Becky Durocher, BA; Nathan Mann, BA; Brett Loomis, MS; Shannon Farley, DrPH, MPH

**Acknowledgements:** Michael Johns, PhD; Kevin Konty, MS; Research Triangle Institute, Inc.; Ewald & Wasserman Research Consultants, LLC; Katherine Bartley, PhD, MDiv

**Background**

Chronic diseases are among the leading health problems in the US; nearly half (49.8%) of all adults in the US have at least one chronic disease.^1^ Many chronic diseases are related to the use or consumption of sugary drinks, unhealthy foods, tobacco, and alcohol, which are considered behavioral risk factors that can be modified by individuals.^2^ However, there is growing consensus that in order to reduce chronic disease prevalence, it is necessary to intervene on multiple levels concurrently, extending beyond the individual to address relationships, community and organizational ties, public policy, and sociopolitical ideology [socioecological model].^3,4^ Many of the above-mentioned individually-modifiable risk factors are associated with products that are heavily advertised in the retail environment (i.e., sugary drinks, unhealthy foods, tobacco, and alcohol).^2^ Since advertising influences consumer behavior, a reduction in exposure to such imagery could translate to fewer negative health outcomes in the long run.

There is a well-established practice of excessive placement of advertisements of unhealthy products in communities of color, low-income areas, and in areas where children congregate (e.g., schools and playgrounds).^5-11^ Several studies have explored the relationship between advertising and varying population characteristics. One study examined associations between the content of outdoor advertising and neighborhood ethnic/racial and socioeconomic composition by capturing photographs of ads in several communities in Los Angeles. In all communities, harmful content, including addictive behaviors (alcohol consumption, tobacco use, gambling), violence, unhealthy eating, unsafe environments for women, and inappropriate content for young children, represented at least 24% of outdoor advertising space. Marketing of harmful content was more prevalent in Asian American and Latino communities.^12^ Another study examined associations between outdoor food advertising, obesity, and soda consumption through analysis of telephone survey data on adults in Los Angeles and Louisiana. The survey collected self-reported information on BMI and soda consumption, which was then linked with a database of directly observed outdoor advertisements. Advertisements were located in densely-populated areas and coded for Global Positioning System (GPS) location, ad content (alcohol, tobacco, food/restaurants, and other), and ad format (posters, flyers, flags, banners, etc.). The researchers found that the higher the percentage of outdoor advertisements promoting food or non-alcoholic beverages within an area, the greater the odds of self-reported obesity among its residents, controlling for age, race and educational status.^13^ A third study looked at outdoor advertising density and content in Los Angeles, Austin, New York City (NYC), and Philadelphia within ZIP codes with contrasting area income levels and predominant ethnicity. Research staff used GPS receivers & cameras to capture outdoor ads, the content of which was then coded for food, non-alcoholic and alcoholic beverages, physical activity, entertainment, and other health-related topics. Results showed wide variation in the amount and type of advertising in ZIP codes; upper-income neighborhoods, regardless of neighborhood ethnicity, were protective against most types of obesity-promoting ads. African American neighborhoods had the highest ad density, followed by Latino neighborhoods; White neighborhoods had the lowest ad density.^7^ A study of the retail environment in communities across the US measured the percentage of retailers with external advertisements for any food or beverage, specifically noting healthy items (i.e., fruit and vegetables) and unhealthy items (i.e., regular soda). A significant association was found between household income and prevalence of ads, with higher odds of displaying any ads, price-promotion ads, and soda ads in low-income community stores than in higher-income community stores.^10^

Previous food and beverage marketing studies have also focused specifically on NYC. A 2017 study of unhealthful marketing assessed content of ads placed on subway platforms at all stations in the Bronx, NYC. Data collectors noted the content (more-healthful vs. less-healthful products), size, language, and ostensible youth- or minority-targeting of food and beverage ads. Advertising density was assessed against demographic information of the area surrounding each station, and findings showed that total ads were more prevalent in areas with higher rates of poverty and higher percentages of children, Black and Hispanic populations, and adults without a high school education.^11^ Finally, a study of outdoor advertising in NYC was conducted to compare the mean number of alcohol-promoting ads across three income levels. The researchers counted the number of alcohol advertisements on retail-dense blocks, then compared the mean number in low-, medium-, and high-income neighborhoods. No significant differences were found among the sampled ZIP codes, but the researchers encouraged further study with power to detect differences at a larger scale.^14^

Although many of the above-mentioned studies support the conclusion that predatory marketing of harmful content is disproportionately found in communities that are predominately of color and/or of low socioeconomic status, there is no gold standard for collecting these data. This document describes a rigorous and useful approach for collecting data on outdoor, street-level advertising of consumable products in an urban environment by neighborhood poverty level.

**Methods**

The density of outdoor advertising in NYC, overall and in low-, medium-, and high-poverty neighborhoods, was measured for: (1) non-alcoholic beverages (including sugary drinks, low-calorie drinks, water/seltzer and other beverages); (2) food products (including fresh produce, fast food, sweets, and other food); (3) tobacco products (including tobacco-containing products as well as electronic nicotine delivery systems (ENDS) such as e-cigarettes); and (4) alcoholic beverages. These four product categories will hereafter be referred to as “consumable products.” The density of outdoor advertising was defined as the number of advertisements per street segment on both sides of the street for these target products, both citywide and in low-, medium, and high-poverty neighborhoods.

In order to define our sampling frame, we began by creating precise parameters by which to measure each of our constructs, including defining “retail-density,” neighborhood poverty, and advertising, and determining protocol for coding images. The procedure and rationale for each of these are described below.

**Defining “Retail-Density”**

We hypothesized that street-level advertisements for consumable products would generally be found on or near retailers that sell such products, and that such areas would draw the most pedestrian traffic, resulting in high exposure to such ads. Therefore, with the aim of finding high advertising density, our goal was to sample “retail-dense” areas; this required defining what geographic units we would sample and how we would designate these units as retail-dense.

We modeled our approach on a previous study of outdoor advertising in NYC, which sampled retail-dense city blocks, or “street segments.”^14^ Street segments consisted of both sides of a street from one cross street to the next, including streets wide enough to be separated by a median. An example of an NYC-specific street segment would be 34th Street between 7th and 8th Avenues. This street segment includes establishments on both the north and south sides of 34th Street. Street segments were designated as retail-dense if 50% or more of building entrances on the street segment contained retail.^14^

Supplementary Table 1 summarizes the six key steps required to identify retail-dense street segments and then code consumable product images. In order to identify street segments, we used a dataset containing information on all street features in NYC, known as Linear Integrated Ordered Network (LION) data. Using geographic information system (GIS) software, LION data were used to analyze all street segments in NYC.^15^ To establish whether street segments were retail-dense, i.e., at least 50% of the segment designated as retail, we used the Primary Land Use Tax Lot Output (PLUTO) dataset Planning.^16^ PLUTO is a land use and geographic dataset with detailed information about individual tax lots, including retail area, census tract, ZIP code, political district (i.e., NYC Community District and City Council District), police and fire precinct, building class, and other information. The data are updated quarterly by the NYC Department of City Planning.

PLUTO data has a designation measuring “floor area, retail,” which estimates the “exterior dimensions of the portion of the structure(s) allocated for retail use”.^17^ Buildings with any ground-floor square footage designated as retail space were considered to be “retail” for this analysis. These buildings typically include the following types of retail establishments: one-story or multi-story retail buildings, department stores, standalone food establishments (i.e., restaurants, grocery stores, and supermarkets), shopping centers and malls, retail bank branches, standalone big box stores, and other miscellaneous retail stores that do not fall into these other categories.^17^

Rather than simply counting each building tax lot, we decided to count the number of doorway entrances within a given street segment to determine the proportion of retail space in a street segment.^18^ The rationale to use doorways instead of tax lots as the unit of analysis was twofold: (1) doorways were likely a more accurate approximation of the number of stores in the retail environment, because tax lots could be divided into several retail spaces and tax lots could vary widely in size, and (2) doorways were easier to accurately assign to street segments in GIS*,* since they were automatically matched with the street segment that they were facing (as opposed to tax lots, which could be problematic to assign if located on a corner).

To identify the building doorways in a retail tax lot, an intermediary GIS dataset of building footprints was used to assign the building identification number (BIN) to each doorway.^19^ Using the BIN, each doorway was designated to be retail or non-retail based on the tax lot associated with the BIN. For tax lots on block corners, doorways were assigned to the street segment that the doorway actually faced, regardless of proximity to one cross street over another.

**Supplementary Table 1. Data sources required to identify retail-dense street segments and code consumable product images, Community Marketing Study, New York City, 2015**

|  |  | |  | |  | | |  |
| --- | --- | --- | --- | --- | --- | --- | --- | --- |
|  | **Step** | **Data Source** | | **Description** | | **Purpose** |  |  |
| **Determine retail-dense  street segments** | 1 | Linear Integrated Ordered Network (LION) data^15^ | | Dataset containing information on all street features in NYC | | Used to analyze all street segments in NYC when joined with a map of NYC |  |  |
|  | 2 | Primary Land Use Tax Lot Output (PLUTO) data^16^ | | A land use and geographic dataset with detailed information about individual tax lots, including retail area, Census tract, ZIP code, political district (i.e., NYC Community District and City Council District), police and fire precinct, building class, and other information. The data are updated quarterly by the NYC Department of City Planning | | Used to designate buildings with any ground-floor square footage designated as retail space. These were typically one- story or multi-story retail buildings, department stores, standalone food establishments (i.e., restaurants, grocery stores, and supermarkets), shopping centers and malls, retail bank branches, standalone big box stores, and other miscellaneous retail stores^17^ |  |  |
|  | 3 | Address Points data^18^ | | Dataset containing information on individual addresses and doorway entrances of NYC buildings | | Used to count the number of doorway entrances within a given street segment to determine the proportion of retail vs non-retail space in a street segment |  |  |
|  | 4 | Building Footprints data^19^ | | An intermediary geographic information system (GIS) dataset of building footprints | | Used to assign the building identification number (BIN) to each doorway. Using the BIN, each doorway was designated to be retail or non-retail based on the tax lot associated with the BIN. |  |  |
| **Determine consumable  product images** | 5 | Photos taken at retail-dense street segments | | Photos taken documenting the presence or absence of street-level, stationary advertisements on retail-dense street segments. Mobile phone cameras were used for capturing photographs; GPS coordinates were embedded in photos as they were taken. For each new street segment visited by data collectors, a new album was created with a unique ID number. Ads were photographed in sequence of their positioning on the street segment, with sufficient detail to identify the address and location type of the ad placement (e.g., restaurant, corner store, phone booth, etc.). | | Used to determine the density of advertising for consumable products within retail-dense street segments throughout NYC |  |  |
|  | 6 | Product definitions for coding advertisement contents | | Detailed definitions of consumable products to be coded into appropriate categories. Includes common examples of products. | | Used to determine how to code each image that appeared in photos taken at retail-dense street segments |  |  |

In order to qualify as a retail-dense street segment, at least half of all doorways on a given street segment had to have a retail designation. For example, on a street segment with 10 total doorways, at least five would have to be coded as retail for the segment to be eligible for our sample. In addition, we set a threshold that to qualify as retail-dense, street segments must have had a minimum of four doorways on a block. Otherwise, a segment with as few as one or two total doorways could have been coded as retail-dense. The minimum threshold of four total doorways would require at least two to be retail establishments and would allow us to maintain **
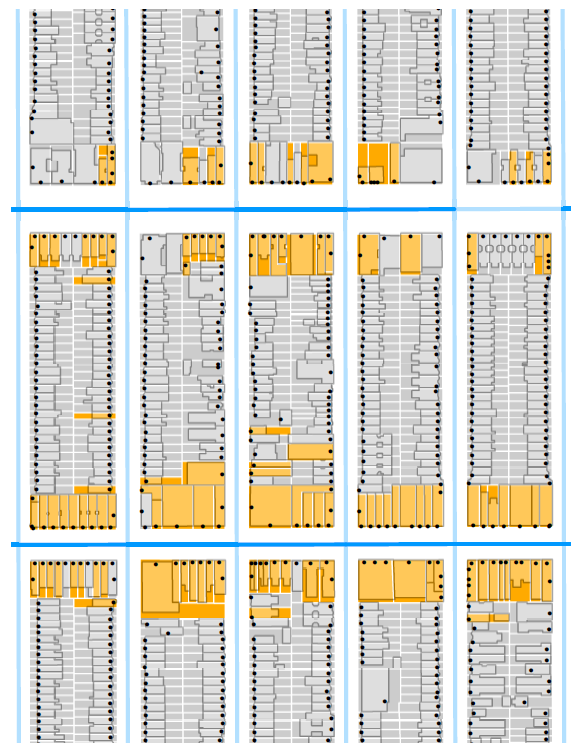
**the capability to reach the desired sample size in each stratum. Supplementary Figure 1 illustrates an example of several cross-streets and their designations based on these criteria.

**Supplementary** **Figure 1. Aerial view of retail-dense street segments in NYC, Community Marketing Study, 2015**

Notes:

Yellow squares are zoned as “retail lots.” Lots vary in size and can have one or more doorway entrances (shown here as black dots).

Light and dark blue lines represent street segments, which include both sides of the street from one cross street to the next.

Dark blue lines are retail-dense. In order to be retail-dense, street segments had to be at least 50% retail on both sides of the street combined (each doorway on a retail lot was coded as “retail.”)

**Defining neighborhood poverty**

The New York City Department of Health and Mental Hygiene (Health Department) provides guidance on how to define neighborhood poverty.^20^ Using these guidelines, along with the US Census Bureau’s American Community Survey 5-year estimates ,^21^ we grouped street segments into three levels of neighborhood poverty based on the census tract that the street segment was contained within: low-poverty, where fewer than 10% of residents live below the federal poverty threshold, medium-poverty, where 10 to <20% of residents live below the threshold, and high-poverty, where 20% or more of residents live below the threshold. Any street segments that bordered census tracts with two different poverty levels were assigned to the higher-income poverty level group. This decision was considered to be more conservative and work against our hypothesis, given that we expected to find higher advertising density in lower-income areas.

**Sampling frame**

A random sample of street segments was selected from eligible retail-dense segments, stratified by neighborhood-level poverty (low, medium, and high) and NYC borough (the Bronx, Brooklyn, Manhattan, Queens, and Staten Island). The target sample size was 1,050 street segments in the 15 strata (five boroughs x three poverty levels). Proportional sampling was used to randomly sample street segments within each stratum, ensuring that each had a least 50 street segments to allow for comparisons between strata; remaining street segments were distributed proportionally into strata. In addition, we oversampled each stratum by 5%, giving us a total of 1,106 street segments in the original sample, with the goal of at least 1,050 total eligible segments; this was done in case segments were ineligible upon visiting, i.e., if they had no stores/retail doorways (e.g., map data were outdated), they were at least 50% covered by scaffolding, the stores/retail doorways were offset from the sidewalk, making the advertisements not visible to passersby, or the segments were otherwise inaccessible.

**Defining Advertisements**

Because there are so many types of marketing and broad parameters within which advertisements might fit, our definition for advertisements was informed by a variety of sources. Advertisements included in this study were street-level, stationary signs (posters, stickers, decals, etc.)^7,9,13^ that displayed a product with the intended purpose of promoting that product or type of product.^22,23^ The discrete, physical unit of the poster, sticker, decal etc., was considered to be a single ad, even if it contained multiple images, except in cases where the images in an ad crossed window panes. In those cases, the entire image was considered to be one ad.

After visiting several retail-dense street segments and photographing a variety of advertisements, we created a more specific and precise list of criteria of what should be included and excluded. Supplementary Table 2 explains the inclusion and exclusion criteria, as well as the rationale behind each of these decisions.^7,9,13,14^

**Supplementary Table 2. Advertisements included in and excluded from the Community Marketing Study, New York City, 2015**

| **Included Advertisements** | **Description** | **Rationale** |
| --- | --- | --- |
| **Content** | | |
| Branded^9^ and non-branded ads | Ads with a nationally- or locally-recognizable logo were considered “branded;” ads with no logo were non-branded. | Both ad types had the intended purpose of promoting a product/type of product or group of products. |
| Ads with logos but without products featured | Ads with logos only were counted as ads for the primary product made by that company. | Logos for companies or products evoke recognition of those products; they have the same intended purpose of promoting purchase of that product, even though the product is not featured. |
| Ads on restaurants with photos of food from the menu | Ads that showed photos of menu items were included, even if showing prices, as long as it was not the complete menu. | These images were intended to promote menu items, which met our definition for advertisements. |
| **Location** | | |
| Ads on stationary, fixed structures^14^ | Ads located on storefronts, awnings, building walls, construction walls, newsstands, bus shelters, subway entrances, etc. were counted. | Ads were included if they were not mobile. These were likely to be part of the environment at all times. |
| Banners tied to the exterior of stores | Ads on banners tied to stores that did not appear to be removed during off-hours. | Banners were considered to be stationary, since it was unlikely that they were moved or removed on a daily basis. |
| Ads located inside an establishment but intended to be seen from the street^14^ | These indoor ads were close enough to the window that customers could not walk between them and the window. Examples included neon signs for alcohol, decals inside a store window facing the street, advertising in window displays, etc. | These ads were meant to be seen from the exterior of the store; therefore, they were considered among the “outdoor” advertising included in the study. |
| Digital ads on subway entrances or other locations | Ads that were displayed on a screen; these frequently rotated a series of ads. | These ads were permanently fixed, despite being rotated. They had the same content as print ads. Data collectors photographed only one ad out of the rotation, whichever was displayed when the photo was taken. |
| **Excluded Advertisements** | **Description** | **Rationale** |
| **Content** | | |
| Ads that primarily promote other products, but also feature food, beverages, or tobacco products. | These ads may unintentionally promote products from our categories of interest, but those products are not the main items promoted by the ad. (e.g., a can of beer found in an ad for a movie). | These ads may include our products of interest, but are not purposely promoting these products; they are promoting other products. |
| Symbols, words, or logos used mainly for store identification ^7,9^ | These images were not counted if they were on the premises of the establishment with which they were affiliated. | These images were not intended to promote a specific product, but rather to identify a store or establishment. |
| Signs that list products but do not feature logos or images | Many stores featured awnings and window displays that listed products they sold, such as the words, “beer,” “candy,” or “cigarettes” | These did not feature a logo or product image, but rather they were informative of products sold by the store. |
| Signs for which less than 50% of the ad is visible/intact | Ads that were mostly damaged, covered, or faded were excluded. | These were considered to be ineffective as ads, since they were difficult to see. |
| Logos for restaurant-affiliated services | Ads for such companies, including rating or delivery services, were excluded unless the ad contained images of food. | These ads were promoting a service that was not specific to a certain food product, unless a product was featured. |
| **Location** | | |
| Non-stationary outdoor ads | Ads found on food carts, sandwich boards, flags, umbrellas, vehicles, etc. | Non-stationary ads were likely to move during the course of the study; in order to limit the parameters of what could be measured, any mobile ad was excluded. |
| Ads located inside an establishment but not intended to be seen from the street | Ads that could be seen through a window, but were far enough from the window that customers could walk between them and the window, or packaged products stacked/shelved in a window. | These ads were not necessarily meant to be seen from the exterior of the store; therefore, they were not considered among the “outdoor” advertising included in the study. Products stacked or shelved in a window were not considered to be intentional advertising, but rather product storage. |
| Ads affixed to/behind doors | These ads are intended to be seen from outside, but could not be clearly seen because the door is propped open. | These ads were excluded because they were partially or completely obstructed from view. |

**Pilot Study and Data Collection**

In advance of data collection, we conducted a pilot study to test and refine the protocol and prepare for previously unanticipated issues with logistics or equipment. The study team and data collectors walked several street segments to observe retailers, grasp the concept of street segments and cross streets, identify ads, emphasize features of advertising of consumable products, and take practice photos. During this session, data collectors also received both classroom and field training. Classroom training led by the study team provided an overview of the project, explained retail-dense street segments, advertisements and consumable products, inclusion and exclusion criteria, how to use equipment and take photos, how to handle challenges, and safety tips. Field training allowed for a hands-on experience taking photos, with teams of two data collectors taking photos along the same street segments and comparing their results with study staff. Six data collectors were ultimately involved in collecting data for the main study.

**Study**

Data collection occurred on both weekdays and weekends between June 22^nd^ and July 22^nd^, 2015, between 11:00am and dusk each day to ensure that businesses that had street-level advertising were open and their storefronts were visible. Mobile phone cameras were used for capturing photographs; GPS coordinates were embedded in photos as they were taken. For each new street segment visited by data collectors, a new album was created with a unique ID number. Ads were photographed in sequence of their positioning on the street segment, with sufficient detail to identify the address and location type of the ad placement (e.g., restaurant, corner store, phone booth, etc.). Multiple ads could be captured within each photograph, as long as sufficient detail of the wording and content of the ad was visible. Digital ads were captured if the content featured consumable products; if they cycled through multiple advertisements, only one out of the rotation was photographed.

Photographs were taken of “street-level” advertisements only; these could be seen by pedestrians walking down the sidewalk on a given street segment. To quantify this measure, data collectors used the top of the first floor, i.e., up to the top of awnings, as their guide for what to consider to be street-level. Signs, banners, etc. above the first floor-level were excluded. If establishments along the street segment were set back from the street, only advertising which was clearly visible and legible from the sidewalk was captured.

Data collectors were provided with instructions on how to respond if they were approached by store owners or bystanders. If a business operator asked them to stop taking photos of their business, they were to stop and record that location as a refusal. If a bystander inquired about data collection, they were to explain that they were taking photographs for a project to learn more about outdoor advertising, clarifying that all ads on selected blocks would be included, and that no photographs of people or information about specific businesses were being collected.

**Advertisement Coding**

A team of coders coded all ads under Health Department guidance; coders received a detailed classroom-style training, they were supervised by study staff throughout the coding process, and they met frequently to discuss and decide how to handle complicated ads. Each advertisement was coded for its location (e.g., store, bus shelter, etc.) and content, including notations for multiple product images within the same ad. A detailed protocol was developed and amended on an ongoing basis as new products and ads were found. Coders made notations for the following designations: (1) non-alcoholic beverages, including sugary drinks, low-calorie drinks, water or seltzer, coffee, other drinks, and unknown drinks; (2) food, including fast food (both from chain and independent restaurants), fresh produce, sweets, and other (any food that did not fall into one of these categories); (3) tobacco, including cigarettes and tobacco paraphernalia, such as rolling papers, hookah pipes, etc., and electronic nicotine delivery systems (ENDS), including e-cigarettes, vape pens, and items included in ENDS kits; (4) alcoholic beverages, including beer, wine, wine products, alcopops, malt beverages, malt liquor, hard liquor, etc.; (5) branded products, with familiar and widely-recognized logos; (6) child-directed marketing, featuring cartoon characters, popular movie, TV, or sports figures, or other deliberate appeal to children;^24,25^ (7) violent or degrading imagery, featuring threatening or sexual treatment of people ;^12,26,27^ and (8) Health Department advertising, featuring public service messages related to consumable products. Detailed definitions and examples of each of these coding designations are presented in Supplementary Table 3.

|  |  |
| --- | --- |
| **Product Type** | **Definition** |
| **Non-alcoholic Beverages** |  |
| Sugary Drinks | Drinks that have added caloric sweetener and greater than 25 calories per 8-ounce serving. |
| Carbonated soft drinks | Sweetened beverages that contain carbonated water and flavoring (commonly known as soda). Widely sold varieties/flavors include cola, lemon-lime, orange, ginger ale, and root beer. |
| Energy drinks | Drinks marketed as a means of boosting energy, decreasing feelings of tiredness, and enhancing mental alertness.^28^ Ingredients often include caffeine, taurine, guarana, ginkgo biloba, carnitine, creatine, additional vitamins or herbs. |
| Sports drinks | Drinks containing added sugar and usually electrolytes, minerals, vitamins, and other nutrients. They are marketed as a means of improving athletic performance by replacing electrolytes and fluid lost in sweat during and after intense physical activity.^28^ |
| Iced tea | A form of sweetened cold tea. Iced teas have the word “tea” on the label. Sometimes mixed with another beverage, such as lemonade. |
| Fruit drinks | Sweetened fruit-flavored drinks, other than sodas, sports drinks, or iced teas. These include -ades, punches, fruit cocktails, juice drinks, etc. |
| Powders and Concentrates | Drinks that require added liquid before consumption. May be powdered, frozen, or in drop form. May also require added sugar or other caloric sweetener. |
| Sweetened Coffee Drinks | Hot and cold coffee beverages, both pre-packaged and made-to-order. Made with sugar or other caloric sweetener and with over 25 calories per 8-ounce serving. |
| Other sugary drinks |  |
| Dairy-based | Sugary drinks that do not fall into above categories and contain dairy products (includes soy).^29^ |
| Non-dairy | Sugary drinks that do not fall into above categories and do not contain dairy products. |
| Low-Calorie Drinks | Drinks that have 25 calories or fewer per 8-ounce serving and include the counterparts to the sugary drink types. |
| Carbonated soft drinks | Same as “Carbonated soft drinks,” but with 25 calories or fewer per 8-ounce serving. |
| Energy drinks | Same as “Energy drinks,” but with 25 calories or fewer per 8-ounce serving. |
| Sports drinks | Same as “Sports drinks,” but with 25 calories or fewer per 8-ounce serving. |
| Iced tea | Same as “Iced tea,” but with 25 calories or fewer per 8-ounce serving. Can be unsweetened. |
| Fruit drinks | Same as “Fruit drinks,” but with 25 calories or fewer per 8-ounce serving. |
| Powders and Concentrates | Same as “Powders and Concentrates” but with 25 calories or fewer per 8-ounce serving. |
| Other low-calorie drinks, non-dairy | Same as “Other sugary drinks - non-dairy” but with 25 calories or fewer per 8-ounce serving. |
| Water/Seltzer | Plain Water: No flavoring, sweetener, carbonation, vitamin additive, etc. Water labels often contain the words “spring,” “filtered,” “pure,” or “purified". Flavored Water: Has flavoring, but NO sweetener, carbonation, or vitamin additive, etc.  Plain Seltzer: Carbonated water with NO flavoring or sweetener, sometimes called “club soda” or “mineral water”. Flavored Seltzer: Carbonated water that has flavoring but NO sweetener, sometimes called “club soda” or “mineral water”. |
| Other Coffee drinks |  |
| Unsweetened | Unsweetened coffee drinks; cups of black or milky coffee are unsweetened unless they are coffee products that are typically sweetened. |
| Unknown | Coffee drinks for which it cannot be determined if they are sweetened or not. |
| Other beverages | 100% juice, milk, hot tea, protein shakes/meal replacement beverages (including powders with nutrition facts) and other beverages not covered above. |
| Unknown beverages | Beverages for which beverage category cannot be determined. |
| **Food** |  |
| Fast Food | Food from chain or independent fast food restaurants, defined as restaurants with a common menu above the counter and no wait staff. Customers typically pay before eating and choose and clear their own tables.^30^ |
| Fresh Produce | Whole, unprocessed fresh fruits and vegetables. |
| Sweets | Sweet snacks and desserts, including cakes, pies, cupcakes, cookies, brownies, dessert bars, ice cream, frozen yogurt, puddings, mousses, gelatins, and candy. Excludes foods intended to be consumed as a meal, e.g. breakfast pastries, donuts, cereal, etc.^31^ |
| Other | Any foods not covered in the categories above. |
| **Tobacco** |  |
| Tobacco & Related Products | Items that contain tobacco which can be smoked, chewed, or otherwise consumed and related tobacco products such as rolling papers, pipes, and hookahs. |
| Electronic nicotine delivery systems (ENDS) & Related Products | Electronic nicotine delivery systems, i.e. electronic cigarettes (e-cigs, e-cigarettes) or personal vaporizers. |
| **Alcohol** | Beverages containing alcohol, including beer, wine, wine products, alcopops, malt beverages, malt liquor, hard liquor, etc. |
| **Branded** | Ads that feature any branding or have any products with a brand name. Brand names are defined as those given by a product manufacturer, which are usually trademarked, and familiar or widely known. |
| **Child directed** | Ads that feature cartoon characters, movie, TV, or sports figures, and ads of kids’ meal toys,^24^ as well as ads depicting “fun appeal” with children laughing, smiling, giggling, or playing, or “action appeal” with excitement or energy associated with a product through children running, jumping, or playing sports.^25^ |
| **Violent or degrading** | This code was be applied in reference to treatment of people (as opposed to animals or fictional creatures). These terms specifically refer to:   Violent Imagery or Language: Depicts the intentional use of physical force or power, threatened or actual, against oneself, or against others that either results in or has a high likelihood of resulting in injury, death, psychological harm, maldevelopment, or deprivation.^27^  Depicts adversarial, abusive, or violent relationships or situations.^26^  Degrading Imagery or Language: Degrades, demeans, or objectifies the form, image or status of any person or group of people. |
| **NYC DOHMH ads** | Ads that are produced by the NYC Department of Health and Mental Hygiene and relate to consumable products. |

When ads contained multiple products, the number of distinct types of products advertised was counted. Identical products that appeared multiple times in the same ad were not counted as multiple products. For example, a six pack of beer, two identical hamburgers, and two identical sodas each counted as one product advertised. If different varieties of one type of product appeared in an ad, including different sizes or flavors of packaged products, each of these counted as a distinct product. Because the same variety/type of fresh produce or other non-packaged items could vary in shape/size, these did not need to be counted as distinct products. If it could not be determined whether repeated product types were exactly the same (such as sandwiches or pizzas), they were counted as separate types of products.

When advertisements consisted of logos only (without any product image or representation), the relevant subcategory was tallied and recorded in the database for each logo. Some logos represent a variety of products rather than just one; in these cases the product for which that company was *most* known was coded. Ads and images were excluded from the database if the products advertised were either less than half visible, or if the product in the image was wrapped in a package without identifying information (e.g., a sandwich in an opaque wrapper and no label).

Ads were also coded for location on a variety of retail establishment types: bodegas or corner stores, supermarkets, smaller food markets selling both groceries and prepared foods, liquor stores, smoke shops, pharmacies or drug stores, gas stations and convenience stores, restaurants (specifying if fast food), confectionary stores, and other stores. Ads not located on retail establishments were coded for these other locations: walls (including temporary construction walls), phone booths, bus shelters, subway entrances, newsstand kiosks or storefronts, and other structures. In addition to documenting location, each ad type was categorized as either a poster/sticker/sign, awning, banner, neon light, or other.

**Results**

A total of 7,963 street segments across NYC qualified as retail-dense; the final sample of 1,106 included 953 eligible and 153 (14%) ineligible segments. Segments were ineligible if upon visiting, they had no stores/retail doorways (n = 98), they were at least 50% covered by scaffolding (n = 31), the stores/retail doorways were offset from the sidewalk, making the advertisements not visible to passersby (n = 14), or the segments were otherwise inaccessible (n = 10) (Supplementary Figure 2). Because of proportional sampling into strata, there was not an exactly even distribution of street segments in each poverty level; however, the original sample had close to one-third of street segments in each poverty level (33% in low poverty, 32% in medium poverty, and 35% in high poverty). The final sample had a similar distribution, with 32% in low poverty, 33% in medium poverty, and 35% in high poverty areas.

**Supplementary Figure 2. Composition of street segments sampled, NYC Community Marketing Study, 2015**


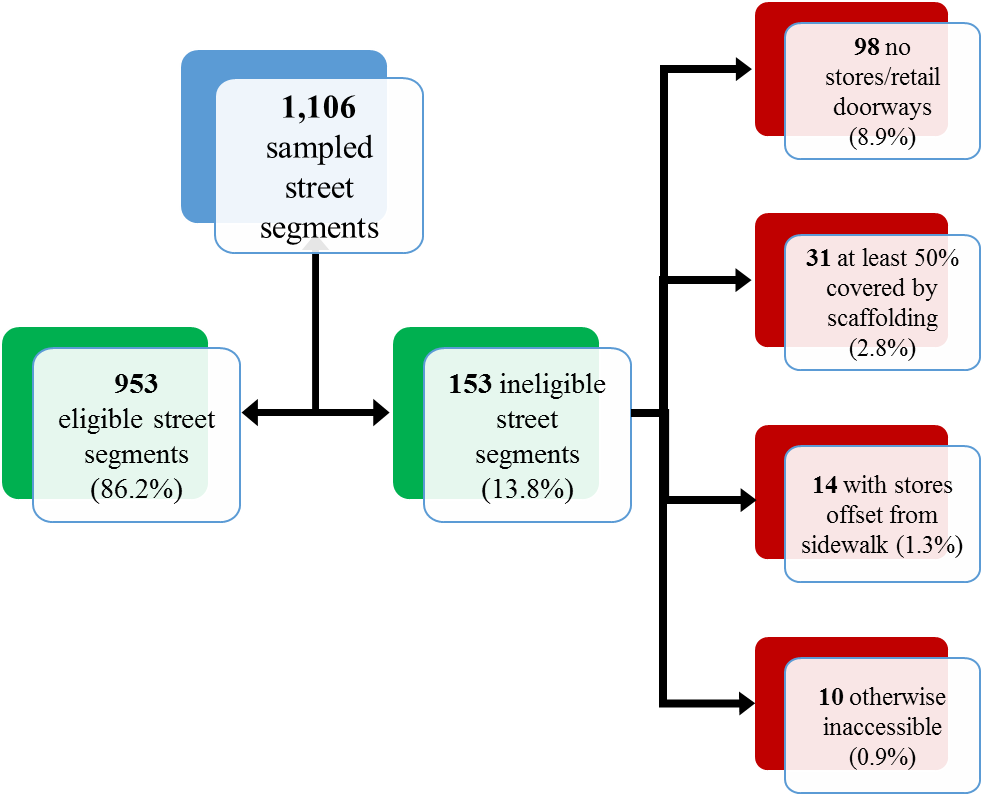


There were a total of 16,305 discrete advertisements for consumable products. The average number of ads per street segment was 17, with a range from 0 to 150. Advertisements were not equally distributed by poverty level, with 42% of all advertisements found in high poverty neighborhoods, 35% in medium poverty neighborhoods, and 24% in low poverty neighborhoods.

Although all eligible street segments had to have at least 2 retail doorways, not every segment had advertising for consumable products (n = 142 eligible segments with no ads, 13% of total sample). A significantly higher percentage of segments in low-poverty neighborhoods had zero advertisements than those in medium- or high-poverty neighborhoods (21% in low-poverty, 13% in medium-poverty, and 11% in high-poverty, p =.005 between low and medium; p=.001 between low and high). Almost half of all ads were located on a bodega or corner store (49%), followed by restaurants (25%), supermarkets (7%), and liquor stores (6%). The vast majority of ads were either posters, stickers, or signs (88%), followed by awnings (5%), neon lights (5%). Banners and “other” made up the remainder (3%).

A total of 50,673 product images were coded in this study across the 16,305 advertisements. The average street segment was 314 feet long (approx. 96 meters), and the average number of images per segment was 53, with a range from 0 to 536. Similar to discrete advertisements, product images were not equally distributed by poverty level, with 44% of all images found in high poverty neighborhoods, 33% in medium poverty neighborhoods, and 23% in low poverty neighborhoods. Aside from displaying content of consumable products, several characteristics were documented at the advertisement level, as described in the manuscript. Supplementary Table 4 presents data in detail.

**Supplementary Table 4. Additional Advertisement Characteristics, NYC Community Marketing Study, 2015**

|  | **Overall** | | **High Poverty** | | **Medium Poverty** | | **Low Poverty** | |
| --- | --- | --- | --- | --- | --- | --- | --- | --- |
|  | **N** | **%** | **N** | **%** | **N** | **%** | **N** | **%** |
| **Advertisement Feature** |  |  |  |  |  |  |  |  |
| Branded products | 11399 | 69.9 | 4725 | 69.6 | 4065 | 72.1 | 2609 | 67.3 |
| Degrading or violent imagery | 583 | 3.6 | 293 | 4.3 | 181 | 3.2 | 109 | 2.8 |
| Public service messaging | 39 | 0.2 | 28 | 0.4 | 6 | 0.1 | 5 | 0.1 |
| Child-focused | 48 | 0.3 | 12 | 0.2 | 20 | 0.4 | 16 | 0.4 |
| **Advertisement Location** |  |  |  |  |  |  |  |  |
| Corner store | 7924 | 48.6 | 3483 | 51.3 | 2752 | 48.8 | 1689 | 43.5 |
| Restaurant | 4097 | 25.1 | 1535 | 22.6 | 1412 | 25.1 | 1150 | 29.7 |
| Other store^a^ | 1615 | 9.9 | 599 | 8.8 | 551 | 9.8 | 465 | 12.0 |
| Supermarket | 1102 | 6.8 | 595 | 8.8 | 286 | 5.1 | 221 | 5.7 |
| Liquor store | 985 | 6.0 | 356 | 5.2 | 441 | 7.8 | 188 | 4.9 |
| Any structure^b^ | 581 | 3.6 | 222 | 3.3 | 194 | 3.4 | 165 | 4.3 |
| Unknown | 1 | 0.0 | 0 | 0.0 | 0 | 0.0 | 1 | 0.0 |
| **Advertisement Type** |  |  |  |  |  |  |  |  |
| Poster/sticker/sign | 14328 | 87.9 | 6046 | 89.0 | 4841 | 85.9 | 3441 | 88.7 |
| Awning | 756 | 4.6 | 363 | 5.3 | 253 | 4.5 | 140 | 3.6 |
| Neon light | 735 | 4.5 | 210 | 3.1 | 316 | 5.6 | 209 | 5.4 |
| Banner | 186 | 1.8 | 69 | 1.0 | 67 | 1.2 | 50 | 1.3 |
| Other | 300 | 1.1 | 102 | 1.5 | 159 | 2.8 | 39 | 1.0 |

Notes:

^a^Other store includes confectionary stores, gas station/chain convenience stores, pharmacy/drug stores, smoke shops, newsstand storefronts, and any other stores not otherwise categorized

^b^Any structure includes phone booths, newsstand kiosks, bus shelters, subway entrances, construction or other walls, and any other structures

**Strengths and Limitations**

Our study methods had a few notable limitations; first, all datasets that were used to define the sample were not updated with the same frequency, so some data were likely to be outdated while others were more current. Second, any advertisements outside of the “street-level” area (such as indoor, subway, billboards, mobile/non-stationary) were excluded, so not every possible advertisement was documented. Thus, the number of ads that New Yorkers are exposed to is likely underestimated by what is documented in this study. Finally, we did not capture the size of advertisements, only the number and content, so each ad was counted as one unit, regardless of size. However, given that each individual product image was counted separately, this may have provided a proxy for size, since multiple images would not necessarily fit onto a very small ad.

This study had several strengths to note as well. First, the use of PLUTO data enabled us to have very detailed information at the tax lot level. Additionally, our large sample size allows for conducting citywide analyses, as well as comparisons between neighborhood poverty levels. Finally, to our knowledge, this is the first study to look at this issue on a citywide level in NYC, with the inclusion of a range of unhealthy products featured in advertising content (food/non-alcoholic beverage, tobacco, and alcohol).

**References**

1. Ward BW, Schiller JS, Goodman RA. Multiple chronic conditions among US adults: A 2012 update. Prev Chron Dis. 2014;11, e62. doi: 10.5888/pcd11.130389.
2. Grier SA, Kumanyika SK. The context for choice: Health implications of targeted food and beverage marketing to African Americans. Am J Public Health. 2008; 98(9), 1616–1629.
3. Sacks R, Yi SS, Nonas C. Increasing access to fruits and vegetables: Perspectives from the New York City experience. Am J Public Health. 2015;105(5), e29-e37. doi: 10.2105/AJPH.2015.302587.
4. McLeroy KR, Bibeau D, Steckler A, Glanz K. An ecological perspective on health promotion programs. Health Educ Q. 1988; 15(4), 351-377.
5. McGinnis M, Goodman JA, Kraak VI. Food marketing to children and youth: threat or opportunity. Washington, DC: IOM, Food and Nutrition Board; 2006.
6. Yale Rudd Center for Food Policy and Obesity. Sugary Drink FACTS, 2014. Accessed November 4, 2016, from: <http://www.sugarydrinkfacts.org/>; 2014.
7. Yancey CK, Cole BL, Brown R, et al. A cross-sectional prevalence study of ethnically targeted and general audience outdoor obesity-related advertising. Milbank Q. 2009; 87, 155-184.
8. Waddell EN, Sacks R, Farley SM, Johns M. Point-of-sale tobacco marketing to youth in New York State. J Adolesc Health. 2016; 59(3), 365-7.
9. Kelly B, Cretikos M, Rogers K, King L. The commercial food landscape: Outdoor food advertising around primary schools in Australia. Aust N Z J Public Health. 2008; 32, 522-528.
10. Isgor Z, Powell L, Rimkus L, Chaloupka F. Associations between retail food store exterior advertisements and community demographic and socioeconomic composition. Health Place. 2016; 39(2016), 43–50.
11. Lucan SC, Maroko AR, Sanon OC, Schechter CB. Unhealthful food-and-beverage advertising in subway stations: Targeted marketing, vulnerable groups, dietary intake, and poor health. J Urban Health. 2017; 94(2), 220-232.
12. Lowery B, Sloane DC. The prevalence of harmful content on outdoor advertising in Los Angeles: land use, community characteristics, and the spatial inequality of a public health nuisance. Am J Public Health. 2014;104(4), 658-64.
13. Lesser LI, Zimmerman FJ, Cohen DA. Outdoor advertising, obesity, and soda consumption: A cross-sectional study. BMC Public Health. 2013; 13:20.
14. Thihalolipavan S, Goranson C, Heller D. Alcohol advertising visible at the street level in retail-dense areas of NYC: A research report from the New York City Department of Health and Mental Hygiene. Accessed November 29, 2016 from: <https://www1.nyc.gov/assets/doh/downloads/pdf/epi/databrief6_whitepaper.pdf>; 2011.
15. New York City Department of City Planning. LION geodatabase [data file]. Retrieved from <http://www1.nyc.gov/site/planning/data-maps/open-data.page>; 2014.
16. New York City Department of City Planning. PLUTO data [data file]. Retrieved from <http://www1.nyc.gov/site/planning/data-maps/open-data.page>; 2014.
17. New York City Department of City Planning, 2014. PLUTO data dictionary. Retrieved from <http://www1.nyc.gov/site/planning/data-maps/open-data.page>; 2014.
18. New York City Department of Information Technology and Telecommunications. Address points data [data file]. Retrieved from <https://data.cityofnewyork.us/City-Government/NYC-Address-Points/g6pj-hd8k/data>; 2015.
19. New York City Department of Information Technology and Telecommunications. Building Footprints data [data file]. Retrieved from <https://data.cityofnewyork.us/Housing-Development/Building-Footprints/nqwf-w8eh/data>; 2015.
20. Toprani A, Hadler JL. Selecting and applying a standard area-based socioeconomic status measure for public health data: Analysis for New York City. NYC DOHMH: Epi Research Report. 2013;1-11.
21. US Census Bureau. Selected economic characteristics, 2009-2013 American Community Survey 5-year estimates. Accessed March 17, 2015 from: <https://factfinder.census.gov/faces/tableservices/jsf/pages/productview.xhtml?src=CF>; 2013.
22. Advertisement [Def. for English Language Learners] Merriam-Webster Online. In Merriam-Webster. Retrieved November 29, 2016, from: <http://www.merriam-webster.com/dictionary/citation>; n.d.
23. World Health Organization. A framework for implementing the set of recommendations on the marketing of foods and non-alcoholic beverages to children. Accessed November 29, 2016 from: <http://www.who.int/dietphysicalactivity/MarketingFramework2012.pdf>; 2012.
24. Ohri-Vachaspat P, Isgor Z, Rimkus L, Powell LM, Barker DC, Chaloupka FJ. Child-directed marketing inside and on the exterior of fast food restaurants. Am J of Prev Med. 2015; 48(1), 22-30.
25. Connor SM. Food-related advertising on preschool television: Building brand recognition in young viewers. Pediatrics. 2006; 118(4),1478-85.
26. Woodruff, K. Alcohol advertising and violence against women: A media advocacy case study. Health Educ Q. 1996; 23(3), 330-345.
27. World Health Organization. Report of the WHO global consultation on violence and health, Geneva, 2–3 December 1996. Unpublished report. (found in World Health Organization. Global status report on violence prevention 2014 Geneva, ISBN 978 92 4 156479 3); 2014.
28. Park S, Onufrak S, Blanck HM, Sherry B. Characteristics associated with consumption of sports and energy drinks among US adults: National Health Interview Survey, 2010. J Acad Nutr Diet. 2010; 113(1), 112-9.
29. United States Department of Agriculture. All about the dairy group. Accessed November 30, 2016 from: <https://www.choosemyplate.gov/dairy>; 2106.
30. Yale Rudd Center for Food Policy & Obesity. Fast food F.A.C.T.S. 2013: Measuring progress in nutrition and marketing to children and teens. Accessed November 30, 2016, from <http://www.fastfoodmarketing.org/media/FastFoodFACTS_report.pdf>; 2013.
31. New York City Department of Health and Mental Hygiene. New York City food standards: Cafeterias/cafes. Accessed November 30, 2016 from: <https://www1.nyc.gov/assets/doh/downloads/pdf/cdp/nyc-cafeteria-cafe-standards.pdf>
32. New York City Department of Health and Mental Hygiene. Community Health Survey, 2013-15.
33. New York City Department of City Planning. New York City population facts. Accessed December 6, 2016 from: <https://www1.nyc.gov/site/planning/data-maps/nyc-population/population-facts.page>; 2016.
34. US Census Bureau. QuickFacts: Chicago city, Illinois. Accessed December 7, 2016 from: <http://www.census.gov/quickfacts/table/PST045215/1714000>; 2010.
35. US Census Bureau. QuickFacts: Philadelphia city, Pennsylvania. Accessed December 7, 2016 from: <http://www.census.gov/quickfacts/table/PST045215/4260000>; 2010.
36. US Census Bureau. Commuting Characteristics by Sex. 2008-2012 American Community Survey 5-Year Estimates. Accessed March 13, 2017 from: <https://factfinder.census.gov/faces/tableservices/jsf/pages/productview.xhtml?src=bkmk>; 2008-2012.
